# Temperature and pH control on lipid composition of silica sinters from diverse hot springs in the Taupo Volcanic Zone, New Zealand

**DOI:** 10.1007/s00792-014-0719-9

**Published:** 2014-12-17

**Authors:** Gurpreet Kaur, Bruce W. Mountain, Matthew B. Stott, Ellen C. Hopmans, Richard D. Pancost

**Affiliations:** 1 Organic Geochemistry Unit, School of Chemistry, University of Bristol, Cantock’s Close, Bristol, BS8 1TS UK; 2 Cabot Institute, University of Bristol, Bristol, UK; 3 GNS Science, Wairakei Research Centre, Private Bag 2000, Taupo, New Zealand; 4 Department of Marine Organic Biogeochemistry, Royal Netherlands Institute for Sea Research, P.O. Box 59, 1790 AB Den Burg, Texel, The Netherlands

**Keywords:** Lipid, Hot spring, Bacterial diether, Archaeol, Hopanoid, Tetraether

## Abstract

Microbial adaptations to environmental extremes, including high temperature and low pH conditions typical of geothermal settings, are of interest in astrobiology and origin of life investigations. The lipid biomarkers preserved in silica deposits associated with six geothermal areas in the Taupo Volcanic Zone were investigated and variations in lipid composition as a function of temperature and pH were assessed. Lipid analyses reveal highly variable abundances and distributions, reflecting community composition as well as adaptations to extremes of pH and temperature. Biomarker profiles reveal three distinct microbial assemblages across the sites: the first in Champagne Pool and Loop Road, the second in Orakei Korako, Opaheke and Ngatamariki, and the third in Rotokawa. Similar lipid distributions are observed in sinters from physicochemically similar springs. Furthermore, correlation between lipid distributions and geothermal conditions is observed. The ratio of archaeol to bacterial diether abundance, bacterial diether average chain length, degree of GDGT cyclisation and C_31_ and C_32_ hopanoic acid indices typically increase with temperature. At lower pH, the ratio of archaeol to bacterial diethers, degree of GDGT cyclisation and C_31_ and C_32_ hopanoic acid indices are typically higher. No trends in fatty acid distributions with temperature or pH are evident, likely reflecting overprinting due to population influences.

## Introduction

The study of geothermal environments and their microbial inhabitants is crucial to unravelling the origins and diversification of life on Earth and the discovery of life elsewhere in the universe (e.g. Stetter 1996). Geothermal systems are populated by diverse and deeply branching thermophilic and hyperthermophilic bacteria and archaea, occurring as planktonic cells in geothermal fluids, and as biofilms and biomats on the surfaces of, and encased within, mineral deposits. Of particular interest are geothermal silica sinters which form rapidly, through abiogenic and biogenic processes, preserving a chemical signature of the local microbial community. Silica deposits and associated microbiology have been studied in hot springs from diverse settings, including Yellowstone National Park, USA (Jahnke et al. 2001; Guidry and Chafetz 2003; Pepe-Ranney et al. 2012), Krisuvik, Iceland (Schultzelam et al. 1995; Konhauser et al. 2001; Tobler and Benning 2011) and the Taupo Volcanic Zone (TVZ), New Zealand (Jones et al. 2001; Mountain et al. 2003; Pancost et al. 2005, Childs et al. 2008; Kaur et al. 2011a, b).

The diverse and characteristic hydrocarbon structures of lipids have a high preservation potential and are entrained with information on biological diversity, environmental conditions and post-depositional alteration history. Cyclisation and methylation, especially within membrane lipids (and their hydrocarbon derivatives), in addition to structural variations in heterocompounds (e.g. terpenoids) are well preserved throughout geological time, and thus represent high-priority targets for early life and astrobiological investigations (Simoneit 2002). Several workers have utilised archaeal and bacterial lipid distributions and carbon isotopic compositions to characterise communities in a range of microbialites (Pancost et al. 2001; Thiel et al. 2001), and to profile mat-building organisms in geothermal systems (Zeng et al. 1992a, b; van der Meer et al. 2000; Jahnke et al. 2004). Moreover, our recent work confirmed the preservation of a wide range of diagnostic lipid biomarkers in geothermal silica sinters and demonstrated their potential in the reconstruction of geothermal microbiology (Pancost et al. 2005, 2006; Kaur et al. 2008, 2011a, b).

Microbial diversity in geothermal settings is fundamentally controlled by the maximum temperature and pH limits of organisms and the environmental conditions (Brock 1978). Although some bacteria are well adapted for survival at extremes of temperature and pH (Brock 1978; Zeng et al. 1992a, b; Rothschild and Mancinelli 2001), Archaea tend to predominate at high temperatures and low pH (Robertson et al. 2005). There is also extensive evidence that environmental conditions can directly influence lipid biosynthesis (Gliozzi et al. 1983; Rothschild and Mancinelli 2001; Schouten et al. 2007), via homeoviscous and homeoproton permeability adaptations to maintain membrane integrity at environmental extremes (Sinensky 1974; Hazel 1995, Albers et al. 2000). For example, at low pH levels, acidophiles modify their lipid composition (e.g. by incorporating membrane-spanning tetraether lipids, Macalady et al. 2004) to maintain a high pH gradient across the cell membrane and the same cytoplasm pH as their mesophilic relatives. To maintain optimal membrane fluidity at high temperatures, thermophiles adjust the membrane lipid composition, incorporating structural motifs that yield more thermally stable membranes (e.g. saturated and long chain fatty acids, Shen et al. 1970; tetraether lipids with cyclopentyl moieties, Gliozzi et al. 1983). Moreover, the ether bonds in archaeal and some bacterial membrane lipids are more stable since they are less readily hydrolysable, particularly under high temperature and low pH. While the effect of temperature and pH on the lipid composition of cultured microorganisms is well documented, little is known on the effect of such conditions on lipid compositions in complex geothermal communities.

In this paper, we examine the lipids preserved in silica sinters associated with six distinct geothermal areas in the TVZ, and assess the relationship between geothermal environment—specifically temperature and pH—and lipid composition. We hypothesise that different environmental conditions will result in distinct lipid profiles reflecting changes in microbial assemblage and/or homeoviscous adaptations.

## Experimental methods

### Site and sample description

**Fig. 1:**
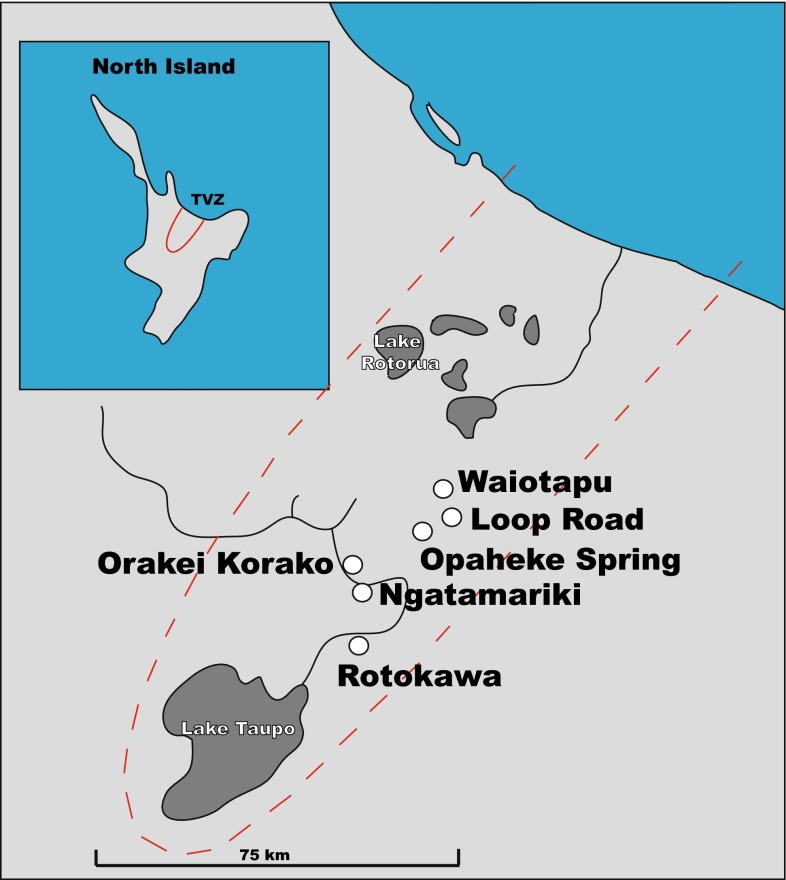
Map showing the six geothermal systems studied within the TVZ

Sinters were collected from six active geothermal systems in the TVZ (Fig. 1). The environmental conditions associated with the analysed sinters are given in Table 1 with further details on the different sites summarised below.

**Table 1:** Conditions associated with the analysed sinters

Location	Sample	Temperature/°C	pH
Champagne Pool	CPa1	75	5.5
	CPa2	75	5.5
	CPa3	75	5.5
	CPa4	75	5.5
	WT1^a^	75	5.5
Opaheke	OP2	90	7.2
	OP3	98	7.2
Loop Road	LRa1	70	5.6
	LRa2	70	5.6
	LRa3	68	5.6
	LRa4	68	5.6
Orakei Korako	OK1	98	7.0
	OK2	83	7.0
	OK3	68	7.0
	OK1D^a^	78	9.0
Rotokawa	RK1a	75	2.8
	RK1F^a^	80	2.5
	RK6A^a^	82	3.7
	RK020211-1	74	2.1
	PK020211-1	81	2.3
Ngatamariki	NGM-49	93	6.7

^a^Samples from previous studies (Pancost et al. 2005, 2006)

#### Champagne Pool

Champagne Pool is located in the Waiotapu geothermal system. The pool water is anoxic and of a mildly acid chloride type, with a pH of 5.5 and a constant temperature of approximately 75 °C. It is supersaturated with respect to amorphous silica (430 mg kg^−1^ SiO_2_; Mountain et al. 2003) and has relatively high H_2_S concentrations. It also contains a wide array of trace elements, including Au, Ag, Sb, W and As (Jones et al. 2001). Active sinters CPa1–CPa4 were collected from the margins of Champagne Pool, at the air–water interface, and are largely composed of elemental sulphur and spicular silica microstromatolites (Kaur et al. 2011a). These samples represent newly formed sinter (<10 year old). Comparisons were made with sinter (WT1) from previous studies (Pancost et al. 2005, 2006). WT1 was also sampled from Champagne Pool and comprised subaqueous domal stromatolite and subaerial spicular sinter (Pancost et al. 2005, 2006).

#### Opaheke Pool

Opaheke spring is located in the Reporoa Caldera situated approximately 6 km south of the Waiotapu geothermal field. These two fields are believed to be hydrologically linked (Nairn et al. 1994). Water temperature here is approximately 98 °C, with a pH of 7.2 and 270 mg kg^−1^ SiO_2_. Two active sinters were collected from the edge of Opaheke Pool, OP2 and OP3. These samples were collected from different regions of the pool, where a small temperature difference is measured (OP2 90 °C, OP3 98 °C). The pH at both sites is 7.2.

#### Loop Road

Loop Road hot springs are situated in a flat, low-lying alluvial plain, a few kilometres south of Waiotapu. LRa1–LRa4 were sampled from active spicular silica sinter from the margins of a Loop Road hot spring. Water temperature is approximately 70 °C and has a pH of 5.6.

#### Orakei Korako

The Orakei Korako geothermal region is approximately 2 km^2^ in area and is located on the eastern margin of the Moroa Volcanic Centre. The waters here are near neutral chloride with high bicarbonate concentrations (Mountain et al. 2003). OK1 was sampled from the edge of Fred and Maggie Pool, whilst OK2 and OK3 were sampled from the outflow channel, where a temperature drop of approximately 15 °C every 10 m was observed. The pool has a temperature of 98 °C and a pH of 7.0. OK1D, discussed in previous studies (Pancost et al. 2005, 2006), originates from Diamond Geyser which has a temperature of 78 °C and a pH of 9.0.

#### Rotokawa

The Sinter Flat area of Rotokawa is a cluster of geothermal springs on the northern margin of Lake Rotokawa that have created a flat terrace, mostly covered in hot pools (Krupp and Seward 1990). Geothermal pools in this region are generally of the acid-sulphate type (Krupp and Seward 1990). Samples were collected from an ebullient hot spring along the northeast margin of the sinter flat. The water temperature was approximately 85 °C in the centre of the pool and dropped to about 60 °C over several metres as the water flowed out in a thin sheet <1 cm in depth. RK1F (80 °C, pH 2.5; Pancost et al. 2005, 2006), composed of spicular silica microstromatolites, was collected from this spring, whereas RK6A (82 °C, pH 3.7; Pancost et al. 2005, 2006) was collected from the south shore of the main upflow zone. The outflow of the Rotokawa hot spring is host to numerous isolated microstromatolites, composed of a multitude of laminations (Mountain et al. 2003). The microstromatolites appear to have grown simultaneously, each from a pumice core, growing outwards and upwards creating coral-shaped structures (Mountain et al. 2003). One of these structures, RK1a (75 °C, pH 2.8), was sampled from the outflow of the hot spring.

Lake Rotokawa drains via Parariki Stream into the Waikato River. RK020211-1 was collected from the western bank of Parariki Stream (water temperature 73.5 °C, pH 2.1), while PK020211-1 was collected from Parariki Spring (81 °C, pH 2.3).

#### Ngatamariki

The Ngatamariki geothermal field is located 15 km north–east of Wairakei and 7 km south–east of Orakei Korako (Fig. 1), covering an area of approximately 10 km^2^. NGM-49 was sampled from a hot spring in the Ngatamariki field. The spring has a temperature of 93.4 °C and a pH of 6.7.

### Lipid analysis

Samples were extracted with dichloromethane (DCM)/MeOH (1:1 v/v) prior to work-up, to remove surface contamination and external biofilms such that the compounds identified likely derived from microorganisms encased in the silica matrix (Pancost et al. 2005). Lipids were present in these extracts, but in lower abundance than in the samples, suggesting that background contamination of the latter was minimal. Samples were dried, ground to fine powder and sequentially extracted by sonication using DCM, DCM/MeOH (1:1 v/v) and MeOH. Activated Cu turnings were added to the extracts, which were left for 24 h to remove elemental sulphur. An aliquot (50 %) of the total extract was fractionated using aminopropyl solid phase extraction (SPE) columns (Phenomenex; NH_2_, 500 mg, 6 ml). The fractions were eluted sequentially with DCM/isopropanol (12 ml, 2:1 v/v; neutral fraction containing hydrocarbons, bacterial diethers, archaeol, GDGTs), 5 % acetic acid in ether (12 ml; acid fraction containing free fatty acids, hopanoic acids) and MeOH (24 ml, polar and inferred phospholipid and glycolipid fraction). Subsequently, 5α-androstane and hexadecan-2-ol (200 ng) were added to the neutral fraction as internal standards. This fraction was then separated into neutral apolar (containing hydrocarbons) and neutral polar (containing alcohols, bacterial diethers, archaeol, GDGTs) fractions by elution through an activated Al_2_O_3_ column with hexane/DCM (9:1 v/v) and DCM/MeOH (1:2 v/v), respectively.

The glycolipid and phospholipid fatty acid components were released by saponification. The acid and polar fractions were heated (1 h) with fresh 0.5 M 95 % methanolic NaOH (1 ml) at 70 °C. The hydrolysed mixture was left to cool and acidified to pH 1–2 with 1 M HCl (ca. 1 ml) and extracted with hexane (3 × 2 ml). The combined extracts were evaporated under N_2_. The fatty acids were methylated using BF_3_/MeOH (100 μl) at 70 °C for 1 h. After cooling, double-distilled water (1 ml) was added. The methyl esters were extracted with DCM (3 × 2 ml) and the combined extracts dried under N_2_. The fatty acid methyl esters (FAMEs) were dissolved in DCM (ca. 1 ml) and eluted through a pre-washed anhydrous Na_2_SO_4_ column to remove residual water. A *n*-C_19_ standard was added and the fractions were dried under N_2_. Neutral polar and methylated acid and polar fractions were derivatised with pyridine (25 μl) and bis(trimethylsilyl)trifluoroacetamide (BSTFA; 25 μl, 70 °C, 1 h) to convert OH groups into trimethylsilyl derivatives.

#### Gas chromatography (GC) and GC–mass spectrometry (GC–MS)

Samples were analysed using a Carlo Erba Instrument HRGC 5300 Megaseries gas chromatograph equipped with a Chrompack CP SIL-5CB column (50 m × 0.32 mm i.d., 0.12 μm film, dimethylpolysiloxane equivalent) and a flame ionisation detector. H_2_ was used as carrier gas, and samples were injected at 70 °C with a temperature programme of 20 °C min^−1^–130 °C and 4 °C min^−1^–300 °C (held 25 min). GC–MS was performed using a Thermo Finnigan Trace gas chromatograph interfaced to a Trace mass spectrometer. The GC column and temperature programme were as above. Electron impact ionisation (70 eV) was used, and full scan spectra were obtained by scanning m/z 50–800 at 1 scan s^−1^. The limits of detection vary amongst compound class but typically range from 10 to 100 ng g^−1^ (dw).

#### Liquid chromatography–MS (LC–MS)

Samples were analysed using high-performance liquid chromatography/atmospheric pressure chemical ionisation–MS (HPLC/APCI-MS) based on a procedure modified from Hopmans et al. (2000) using an Agilent 1100 series/Hewlett-Packard 1100 MSD series instrument equipped with an autoinjector and Chemstation software. Separation was achieved with a Prevail Cyano column (2.1 mm i.d. × 150 mm, 3 μm; Alltech) at 30 °C. Typical injection volume was 10 μl. Glycerol dialkyl glycerol tetraethers (GDGTs) were eluted isocratically with 99 % hexane and 1 % isopropanol for 5 min, followed by a linear gradient to 1.6 % isopropanol for 40 min. Flow rate was 0.2 ml/min. After each analysis, the column was cleaned by back-flushing hexane/propanol (95:5, v/v) at 0.2 ml/min for 10 min. Detection was achieved using positive ion APCI. Conditions for the Agilent 1100 APCI-MS instrument were: nebulizer pressure 60 psi, vaporiser temperature 400 °C, drying gas (N_2_) flow 6 l/min and temperature 200 °C, capillary voltage −3.5 kV, corona current 5 μA. Positive ion spectra were generated by scanning from m/z 900 to 1400.

## Results and discussion

A wide range of lipid biomarkers were detected in the TVZ silica sinters. Key bacterial biomarkers include free fatty acids, inferred 1,2-diacylglycerophospholipids, 1,2-di-*O*-alkylglycerols (diethers), 1-*O*-alkylglycerols (monoethers) and various hopanoids. Dominant archaeal lipids include 2,3-di-*O*-phytanylglycerol (archaeol) and glycerol dialkyl glycerol tetraethers (GDGTs). We focus solely on the abundances and distributions of the aforementioned lipids, which we attribute to bacterial and archaeal origin.

### Sources of lipids

#### Champagne Pool

The likely sources of lipids in the Champagne Pool sinters are discussed in detail in Kaur et al. 2011a. Briefly, bacterial non-isoprenoidal diethers were attributed to *Thermodesulfobacteriales* (*Thermodesulfbacterium hydrogeniphilum*) and *Aquificales* (*Venenivibrio stagnispumantis*) (Table 2), consistent with DNA analyses of Champagne Pool sinters and waters (Hetzer et al. 2007; Childs et al. 2008), whereas archaeol and GDGTs were attributed to *Sulfolobales* (*Sulfurisphaera ohwakuensis*) and/or *Thermofilum*-like populations (Table 2; Hetzer et al. 2007). Low-molecular weight (LMW) fatty acids have a range of potential sources, although branched fatty acids were attributed to *Thermodesulfobacteriales* (Langworthy et al. 1983). Neither *Aquificales* nor *Thermodesulfobacteriales* are known to synthesise hopanoids; instead these compounds have been attributed to a *Bacillales* and *Burkholderiales* source (Gibson et al. 2014).

**Table 2:** Biomarker interpretations for each site

	Sample	Diethers	Macrocyclic diether	Diethers + monoethers	Fatty acids	Archaeol	Hopanoids	Cyanobacterial biomarkers	Chloroflexus biomarkers	Interpretation
Champagne Pool	CPa1-4	+			C_16_, C_18_, C_20_	+	+			*Thermodesulfobacteriales Aquificales*, *Sulfolobales*, *Thermofilum*
	WT1	+			C_16_, C_18_, C_20_	+	+			
Opaheke	OP2,3			+	C_16_, C_18_, C_20_	+	+			*Aquificales*, unknown bacterial input, Crenarchaeota, Euryarchaeota
Loop Road	LRa1-4	+			C_16_, C_18_	+	+			*Aquificales, Thermodesulfobacteriales* unknown bacterial input, Euryarchaeota
Orakei Korako	OK1			+	C_16_, C_18_, C_20_	+	+	+		*Aquificales*, unknown bacterial input, Crenarchaeota, Euryarchaeota Allochthonous cyanobacterial input
	OK1D			+	C_16_, C_18_, C_20_	+	+		+	Same as above but allochthonous *Chloroflexus* input
Rotokawa	RK1a				C_16_, C_18_	+	+			*Aquificales*, Thermoacidophilic *Thermoprotei*
	RK1F	+	+		C_16_, C_18_	+	+			
	RK6A	+	+		C_16_, C_18_	+	+			
Ngatamariki	NGM-49			+	C_16_, C_18_, C_20_	+	+			*Aquificales*, unknown bacterial input, Crenarchaeota, Euryarchaeota

#### Loop Road

The Loop Road spring sinters contain a variety of archaeal and bacterial lipids, which can be used to tentatively define the microbial community structure. The bacterial lipids are characterised by the predominance of diethers, particularly the C_17_/C_18_ and C_18_/C_18_ components, and an absence of monoethers. A variety of fatty acids (including branched, unsaturated and hydroxylated components) and hopanoids, present in a range of stereoisomers, were also identified. Archaeal lipids include archaeol and GDGTs, with a predominance of tetraethers lacking cyclopentyl moieties. This biomarker assemblage is similar to that observed in the active Champagne Pool sinters (Table 2). Indeed, preliminary bacterial DNA analyses of Loop Road spring reveal the presence of *Aquificales* and *Thermodesulfobacteriales* (Stott, personal communication), the main bacterial orders identified in Champagne Pool. The dialkyl glycerol diethers predominant in the Loop Road sinters likely derive from *Aquificales* and *Thermodesulfobacteriales*. The fatty acids can be attributed to multiple sources; however, the LMW branched-chain components (*i*-C_15:0_ and *a*-C_15:0_, and *i*-C_17:0_ and *a*-C_17:0_) are most likely of a thermodesulfobacterial origin (Langworthy et al. 1983), while the LMW unsaturated components (specifically C_18:1_) likely derive from *Aquificales* (Jahnke et al. 2001). Hopanoids are also detected in the Loop Road sinters; their origin remains unclear, possibly deriving from an anaerobic organism, given the anoxic waters, or from an aerobic microbial strain living near the air–water interface. Archaeal lipids in the Loop Road sinters include archaeol and GDGTs. Archaeol is ubiquitous in the Loop Road sinters, suggesting a significant contribution from archaea. The lack of cyclopentane-bearing GDGTs and the predominance of the acyclic GDGT suggest a euryarchaeotal source (Schouten et al. 2007).

#### Opaheke spring

The Opaheke spring sinters are characterised by a predominance of bacterial diethers, particularly the C_18_/C_18_ and C_18_/C_19_ components. Monoethers, fatty acids (including branched, unsaturated and hydroxylated components) and various hopanoids were also identified. Archaeal lipids comprise archaeol and GDGTs, with a predominance of tetraethers bearing 3 or 4 cyclopentane rings.

Dialkyl glycerol diethers are predominant membrane lipids in thermophilic bacteria including the *Aquificales* (Huber et al. 1992), *Ammonifex degensii* (Huber et al. 1996) and *Thermodesulfobacterium commune* (Langworthy et al. 1983). The high temperature of this system is inconsistent with a *Thermodesulfobacteriales* or *Ammonifex degensii* source (Wagner and Wiegel 2008), suggesting an *Aquificales* origin (Table 2). Previous studies reveal different alkyl chain distributions for different *Aquificales* cultures: Huber et al. (1992) reported C_16_/C_16_, C_17_/C_17_ and C_17_/C_18_ as the dominant diethers in *Aquifex pyrophilus* (optimum growth 85 °C, pH 6.8), whereas Jahnke et al. (2001) reported C_18_/C_18_, C_18_/C_20_ and C_18_/C_21:1_ as the main components in a range of *Aquificales* cultures including *Aquifex aeolicus*, *Aquifex pyrophilus* and *Thermocrinis ruber* (85 °C, pH 6.8). The distribution observed here, dominated by the C_18_/C_18_ and C_18_/C_19_ components, appears to fall between these two end members. The presence of additional biomarkers for *Aquificales*, such as monoethers, is consistent with an *Aquificales* origin (Jahnke et al. 2001).

The fatty acids identified in the Opaheke sinters likely derive from multiple sources: high-molecular weight (HMW) components deriving from both plant and bacterial sources and LMW fatty acids (i.e. C_14_–C_20_) deriving from a variety of bacterial sources. LMW unsaturated components such as C_18:1_ can be attributed to an *Aquificales* source (Jahnke et al. 2001), while branched-chain fatty acids, *i*-C_15:0_ and *a*-C_15:0_, and *i*-C_17:0_ and *a*-C_17:0_, can derive from diverse sources including sulphate-reducing bacteria and Gram-positive bacteria (Langworthy et al. 1983; Zelles 1999). β-OH fatty acids are associated with the lipopolysaccharides of Gram-negative bacteria, but can also be present as the fatty acyl components of PLFAs (Zelles 1999). Hopanoids, although present in subordinate abundance, are also detected in the Opaheke sinters; the source of these compounds is unclear. Bacteriohopanepolyols (biological precursors of hopanoids) of inferred cyanobacterial and methanotrophic bacterial origin were previously detected in an inactive sinter from Opaheke spring (Gibson et al. 2008). In this study, the high temperature of the active system is inconsistent with a cyanobacterial source, and the hopanoic acids possibly derive from methanotrophic bacteria.

Archaeal lipids in the Opaheke sinters include archaeol and GDGTs. Archaeol is present in abundances comparable to the other compounds in these sinters, indicating a significant contribution from archaea. The tetraethers are dominated by components bearing 3 or 4 cyclopentane rings. GDGTs with 0–4 cyclopentane moieties are dominant in (hyper)thermophilic *Crenarchaeota* and *Euryarchaeota*, including the crenarchaeotal order *Thermoproteales* (Schouten et al. 2007), whereas those bearing 4–8 cyclopentane rings are relatively uncommon and likely derive from the euryarchaeotal order *Thermoplasmatales* and the crenarchaeotal order *Sulfolobales* (Schouten et al. 2007). Thus, the archaeal GDGT distributions in these sinters are consistent with a range of *Crenarchaeota* and *Euryarchaeota* sources.

#### Orakei Korako

A range of bacterial and archaeal biomarkers were detected in the sinters from Fred and Maggie Pool and its outflow channel. Dominant bacterial biomarkers include free fatty acids, inferred 1,2-diacylglycerophospholipids, bacterial diethers, monoethers and various hopanoids. Archaeal lipids include archaeol and GDGTs. This biomarker assemblage is similar to that observed in the Opaheke sinters (Sect. “Opaheke spring”; Table 2), and can be used to tentatively define the microbial community structure (Table 2). Previous lipid analyses of sinter from Diamond Geyser in Orakei Korako (OK1D), revealed the presence of C_18_, C_20_ and C_20:1_ monoethers, biomarkers inferred to derive largely from *Aquificales* (Pancost et al. 2005, 2006). Members of this order represent bacteria with the highest growth temperatures, with *Aquifex pyrophilus* having an upper growth temperature of 95 °C (Wagner and Wiegel 2008). Indeed, the bacterial ether lipids present here, specifically the C_18_/C_19_ diether and C_18_ and C_20_ monoethers, and the physical conditions at Fred and Maggie Pool are consistent with an *Aquificales* source. The LMW alkanoic acids observed likely derive from a mixing of various bacterial sources. However, C_18:0_, C_18:1_ and C_20:1_ fatty acids have been identified in a variety of *Aquificales* cultures (Jahnke et al. 2001), and in the Orakei Korako sinters these compounds could derive from these organisms, consistent with ether lipid analyses and previous studies (Pancost et al. 2005, 2006). The LMW branched-chain fatty acids and the hopanoic acids, however, likely derive from an alternate bacterial source since *Aquificales* are not known to synthesise these compounds. Indeed, Talbot et al. (2005) detected bacteriohopanetetrol and bacteriohopanepentol in a previous study of OK1D, and attributed these compounds to a cyanobacterial origin.

Archaeal lipids in the Orakei Korako sinters include archaeol and GDGTs. Archaeol occurs in concentrations comparable to those of the bacterial ether lipids, suggesting a significant contribution from archaea, consistent with the high water temperature at this site. In addition, a range of GDGTs was observed, with a predominance of components bearing 0–4 cyclopentane rings, suggesting a euryarchaeotal and/or crenarchaeotal origin; however, the specific sources are unclear (Schouten et al. 2007). Biomarkers for cyanobacteria (e.g. *n*-alkenes and monomethyl alkanes) were also detected, albeit in subordinate abundance. Since the temperature at Fred and Maggie Pool is considerably higher than the maximum growth temperatures for cyanobacteria (73 °C), these biomarkers likely represent an allochthonous cyanobacterial input from the surrounding sinter flat.

#### Ngatamariki

NGM-49 contains a range of archaeal and bacterial lipids. Bacterial lipids include free fatty acids (mostly LMW saturated and unsaturated components), inferred 1,2-diacylglycerophospholipids, hopanoids, monoethers and a suite of non-isoprenoidal diethers. As discussed in Sect. “Opaheke spring”, diethers in the presence of monoethers suggest an *Aquificales* source (Pancost et al. 2006). Members of the *Aquificales* order represent bacteria with the highest growth temperatures (Wagner and Wiegel 2008). The bacterial ether lipids detected here, specifically the C_17_/C_18_ and C_18_/C_18_ diethers and C_18_ and C_20_ monoethers, in addition to the high water temperature at Ngatamariki are all consistent with an *Aquificales* origin. The LMW fatty acids (i.e. C_14_–C_20_) derive from a variety of bacterial sources; however, it is possible that the C_18:0_, C_18:1_ and C_20:1_ fatty acids detected here also derive from *Aquificales* (Jahnke et al. 2001). The hopanoids likely derive from an alternate unknown bacterial source.

Archaeal lipids detected include archaeol and GDGTs. Archaeol is once again present in concentrations comparable to the bacterial ether lipids, consistent with the high temperature at this site. A range of GDGTs were also detected, with a predominance of tetraethers bearing 3 or 4 cyclopentane rings, suggesting a crenarchaeotal and/or euryarchaeotal source (Schouten et al. 2007). This biomarker assemblage is similar to that observed in the physicochemically similar Orakei Korako (OK1) and Opaheke sinters (Table 2).

#### Rotokawa

The Rotokawa stromatolite (RK1a) contains a range of archaeal and bacterial lipids, including free fatty acids, inferred 1,2-diacylglycerophospholipids, various hopanoids, archaeol and GDGTs. In previously analysed sinters, RK1F and RK6A, bacterial non-isoprenoidal diethers and macrocyclic diethers were also detected (Pancost et al. 2005). Preliminary DNA analyses reveal *Aquificales* (*Hydrogenobaculum*) and *Thermoprotei* (*Sulfolobus* and *Sulfurisphaera*) in a variety of Rotokawa hot springs (Stott, personal communication). The bacterial diether lipids in RK1F and RK6A most likely derive from *Aquificales*; the source of the macrocyclic analogues remains unknown, but likely deriving from a thermoacidophilic source (Pancost et al. 2006). The fatty acids identified in the stromatolite likely derive from multiple sources: HMW components deriving from both plant and bacterial sources, consistent with carbon isotopic values recorded by Pancost et al. (2005), and LMW fatty acids (i.e. C_14_–C_20_) deriving from a variety of bacterial sources. Hopanoids were also detected in a range of stereoisomers, albeit in subordinate abundance; the source of these compounds is unclear. The predominance of GDGTs bearing 0–4 cyclopentane rings and the conditions at Rotokawa spring are also consistent with a thermoacidophilic *Thermoprotei* source (Perevalova et al. 2010).

#### Summary

The overall biomarker profiles reveal three diverse assemblages across the TVZ sites, correlating with three distinct geochemical environments: the first in the Champagne Pool and Loop Road sinters (slightly acidic, ~70 °C), the second in the Orakei Korako, Opaheke and Ngatamariki sinters (neutral pH, ≥90 °C), and the third in the sinters from Rotokawa (highly acidic, ~80 °C). This suggests that comparable temperature and pH conditions, even across different settings, result in similar microbial communities and subsequently similar biomarker distributions.

### Biomarker variations with temperature and pH

#### Bacterial diether lipids

**Fig. 2:**
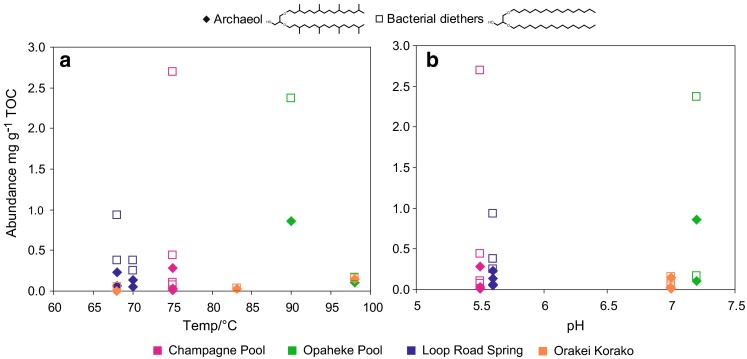
Archaeol and total bacterial diether abundances (mg g^−1^ TOC) versus **a** temperature and **b** pH. *Closed diamonds* represent archaeol abundances and *open squares* represent bacterial diether abundances

A range of bacterial diether lipids comprising straight-chain non-isoprenoidal alkyl components were identified in the geothermal sinters, with their respective alkyl components tentatively identified on the basis of retention times and mass spectra (Pancost et al. 2001, 2006). Non-isoprenoidal diethers have been identified in a variety of marine (Pancost et al. 2001; Bouloubassi et al. 2006) and terrestrial environments, including geothermal sinters from the TVZ (Pancost et al. 2005, 2006) and in Yellowstone National Park (Zeng et al. 1992a, b; Jahnke et al. 2001). Total bacterial diether abundances range from 0.031 in OK2 to 2.7 mg g^−1^ TOC in CPa1 (Table 3; note that for samples RK1a, RK1F, RK6A, RK020211-1, PK020211-1, WT1, OK1D and NGM-49, TOC-normalised abundances are unavailable; distributions are comparable and are discussed subsequently). Total diether lipid concentrations exhibit no correlation with temperature or pH (Fig. 2a, b). If the range of samples is restricted to hot springs with a pH in the range 5.5–7.2, there is still no correlation of diether concentration with temperature. Furthermore, no correlation with pH is apparent when samples are restricted to those within the temperature range of 68–82 °C.

**Table 3:** Abundance (mg g^−1^ TOC) and distributions of archaeol and bacterial diethers at each geothermal site

	Temp/°C	pH	Archaeol	Bacterial diethers	Archaeol/bacterial diethers	ACL
CPa1	75	5.5	0.29	2.7	0.11	17.3
CPa2	75	5.5	0.037	0.44	0.08	17.1
CPa3	75	5.5	0.008	0.074	0.10	17.1
CPa4	75	5.5	0.010	0.10	0.09	17.1
WT1	75	5.5	–	–	0.14	17.0
OP2	90	7.2	0.86	2.4	0.36	17.9
OP3	98	7.2	0.10	0.16	0.63	17.9
LRa1	70	5.6	0.14	0.38	0.37	17.3
LRa2	70	5.6	0.056	0.25	0.23	17.4
LRa3	68	5.6	0.23	0.94	0.24	17.4
LRa4	68	5.6	0.066	0.38	0.17	17.5
OK1	98	7.0	0.15	0.16	0.96	18.5
OK2	83	7.0	0.019	0.031	0.61	18.5
OK3	68	7.0	ND	0.059	0.0	15.7
OK1D	78	9.0	–	–	0.59	18.0
RK1a	75	2.8	–	ND	–	–
RK1F	80	2.5	–	–	3.0	15.7
RK6A	82	3.7	–	–	0.16	15.6
RK020211-1	74	2.1	–	ND	–	–
PK020211-1	81	2.3	–	ND	–	–
NGM-49	93	6.7	–	–	0.41	17.6

*ND* not detected

**Fig. 3:**
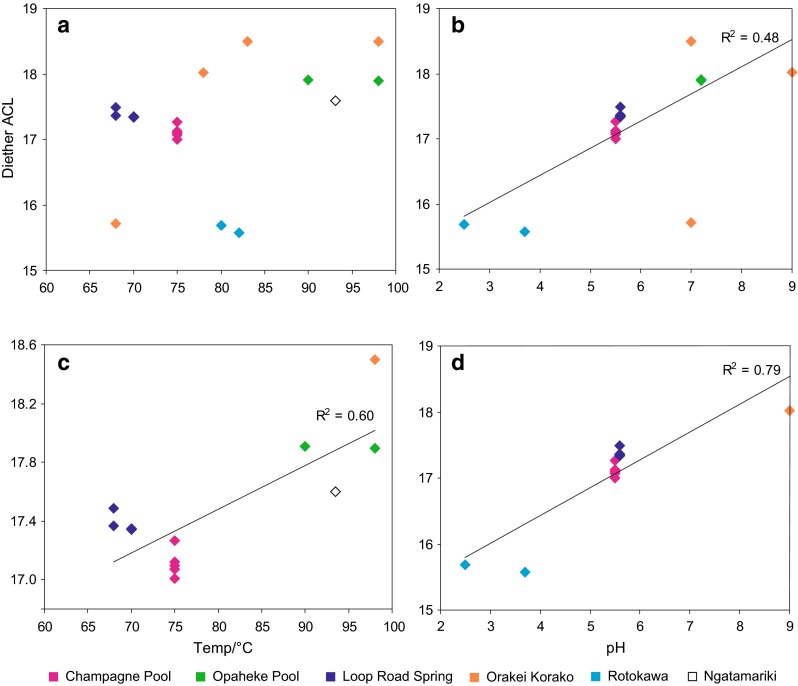
Bacterial diether ACL versus **a** temperature and **b** pH. In **c**, diether ACLs have been plotted against temperature where samples have been restricted to those from hot springs with a pH range of 5.5–7.2; in **d** diether ACLs have been plotted against pH where samples have been restricted to those associated with a temperature range of 68–82 °C

In contrast, relationships between bacterial diether average chain length (ACL) and both temperature and pH are apparent (Fig. 3a–d). For the entire dataset, the correlation between diether ACL and temperature is poor (Fig. 3a). This is partly due to the very low diether ACLs in the Rotokawa sinters (characterised by low pH), high diether ACLs in the Diamond Geyser sinter (characterised by high pH), and the varying diether ACLs from the Orakei Korako outflow channel. Although the latter do increase with temperature, the relationship is different from that of the larger data set (Fig. 3a), perhaps arising from the different hydrological regime—narrow outflow channels with fast flowing waters as opposed to pools. Removing the extreme pH samples and the Orakei Korako outflow samples results in a much stronger relationship (Fig. 3c).

For the entire dataset, a weak correlation between diether ACL and pH is observed (*R* ^2^ = 0.48, Fig. 3b). The outliers comprise a high diether ACL in sinter from Fred and Maggie Pool (characterised by high temperature) and varying diether ACLs in sinters from its outflow channel, likely reflecting the different hydrological regime. Removing the extreme temperature sinters and Orakei Korako outflow samples results in a much stronger correlation between diether ACL and pH (*R* ^2^ = 0.79, Fig. 3d).

Different organisms appear to have significantly different distributions of non-isoprenoidal diethers; for example, Jahnke et al. (2001) reported C_18_/C_18_, C_18_/C_20_ and C_18_/C_21:1_ as the main components in *Aquificales* cultures, while Langworthy et al. (1983) identified C_16_/C_16_, C_16_/C_17_, C_17_/C_17_, C_17_/C_18_, C_18_/C_18_ as the principle diethers in *Thermodesulfotobacterium commune*, although we note that these were also grown under different temperatures (85 and 65 °C, respectively, but the same pH 6.8). Thus, at least some of the observed variation in these sinters could reflect differences in the microbial community. Nonetheless, the correlation of diether ACL with temperature suggests that there could be a partial environmental control. Although little is known about the role of bacterial diethers in homeoviscous adaptation, the acyl chain length of phospholipids has been shown to increase at higher growth temperatures (e.g. Russell 1984), and a similar effect could occur for the bacterial diethers. Higher molecular weight diethers would have higher thermostabilities, helping to maintain optimal membrane fluidity at higher temperatures and this could explain the observed high diether ACL at elevated temperatures.

Previous researchers have also reported an increase in fatty acid acyl chain length at lower pH (Drici-Cachon et al. 1996), and a similar effect might be expected for diether ACLs. However, the opposite is observed in this work, with higher diether ACLs associated with sinters deposited under neutral rather than acidic pH. This could reflect a different role for diether lipids in regulating membrane stability than that played by glycerol acyl lipids; however, it is likely to reflect the different inferred community assemblages in the different pools governing the relationship shown in Fig. 3d.

#### Archaeol

**Fig. 4:**
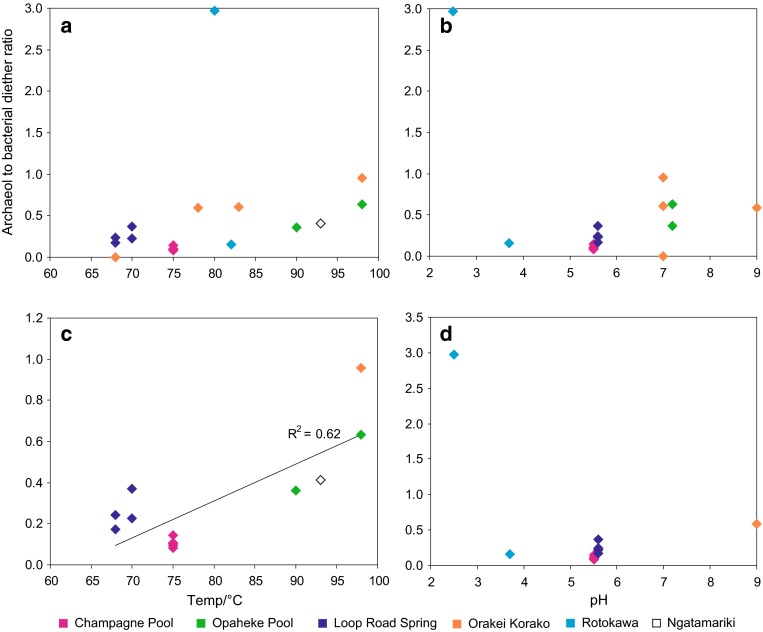
Archaeol to bacterial diether ratio versus **a** temperature and **b** pH. In **c**, ratios have been plotted against temperature where samples have been restricted to those from hot springs with a pH range of 5.5–7.2; in **d**, ratios have been plotted against pH where samples have been restricted to those associated with a temperature range of 68–82 °C

Archaeol is widely distributed amongst archaea (DeRosa and Gambacorta 1988), and has been previously identified in geothermal environments (Ward et al. 1985; Pancost et al. 2005, 2006). The abundance of archaeol in the silica sinters is highly variable ranging from 0.008 in CPa3 to 0.86 mg g^−1^ TOC in OP2 (Table 3). Archaeol is typically less abundant than the bacterial diethers, except in the most acidic sinters (see below). Archaeol concentrations exhibit no trend with temperature or pH (Fig. 2), and similarly no correlation is observed between pH or temperature and archaeol to bacterial diether ratios (Fig. 4a, b; Table 3). However, with respect to temperature, this lack of correlation is largely due to the very high ratio in one acidic Rotokawa sample. If this and other pH extreme samples are excluded, a correlation between the archaeol to bacterial diether ratio and temperature is apparent, with ratios generally increasing with temperature (Fig. 4c).

No strong correlation is observed between this ratio and pH in either the entire or temperature-constrained datasets (Fig. 4b, d); however, the highest concentrations of archaeol relative to the bacterial diethers do occur in the most acidic sinters, RK1F, RK1a, RK020211-1 and PK020211-1 (Table 3). In the latter three, this arises from the absence of non-isoprenoidal diethers. High archaeol abundances and archaeol to bacterial diether ratios were also noted in our previous study for a sinter deposited under acidic conditions at Champagne Pool (Kaur et al. 2011a). Archaea, well adapted to survival at extremes, tend to predominate over bacteria at high temperatures and low pH (Robertson et al. 2005), likely explaining these observations. It is intriguing that these relationships persist even when focussing on a subset of the bacterial population (i.e. diether producers) that are also adapted to environmental extremes. Indeed, Reysenbach et al. (2005) reported the survival of only one genus of *Aquificales* in acidic conditions (*Hydrogenobaculum*), which could account for the high relative archaeol concentrations observed in these sinters.

#### GDGTs

**Fig. 5:**
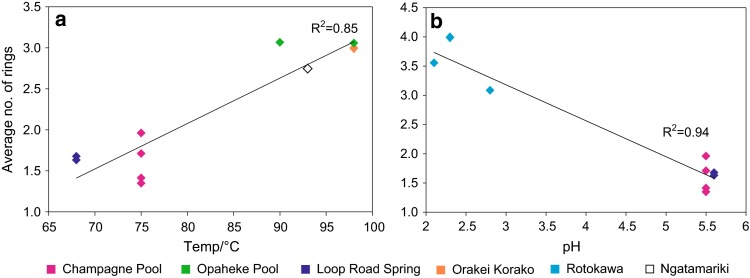
Average number of cyclopentane rings in the GDGT biphytanyl chains versus **a** temperature and **b** pH. For variations with temperature, samples have been limited to those with pH in the range 5.5–7.2; for variations with pH, samples have been limited to those within the temperature range 68–82 °C

Isoprenoid glycerol dialkyl glycerol tetraethers (GDGTs) are the predominant membrane lipids of hyperthermophilic archaea and have been identified in geothermal sinters, mats and sediment (Ward et al. 1985; Zhang et al. 2006; Pancost et al. 2006; Schouten et al. 2007). However, they are not exclusive to these organisms and have also been observed in non-thermophilic environments (Schouten et al. 2000; Weijers et al. 2006). Previous studies reveal that thermophilic archaea respond to high temperature and low pH conditions by increasing the ratio of GDGTs to diethers, and the proportion of GDGTs with cyclopentyl moieties in their membranes (Gliozzi et al. 1983). These components help stabilise the cell membrane, maintaining optimal membrane fluidity and reducing proton permeability (Albers et al. 2000). A selection of sinters was analysed for GDGTs but concentrations were not determined. A range of isoprenoid tetraether lipids, with a total number of cyclopentyl rings ranging from 0 to 8, was identified on the basis of retention times and mass spectra. From previous biomarker classes, it is clear that by constraining the temperature and pH ranges, distribution trends become more apparent, and this is the approach taken for GDGT and subsequent comparisons. GDGT distributions correlate with both temperature and pH (Fig. 5). For a given pH range (5.5–7.2), the average number of cyclopentyl rings in the biphytanyl chains typically increases with temperature (Fig. 5a). Distributions also vary with pH; for a given temperature range (68–82 °C), the average number of cyclopentane rings is significantly higher in the Rotokawa sinters, which precipitated in much more acidic waters (Fig. 5b). These distribution trends could reflect variations in microbial assemblage due to the varying environmental conditions. This is particularly true for the observed pH relationship given the fact that it is largely driven by differences between the Rotokawa sinters from all the others. Biomarker distributions from Champagne Pool and Loop Road suggest similar microbial populations at these sites as do the distributions at Orakei Korako, Opaheke and Ngatamariki, such that the relationships between the number of cyclopentane rings and temperature within those respective groups likely reflect homeoviscous adaptive responses.

#### Fatty acids

Fatty acids are important constituents of bacterial and eukaryotic cell membranes and are, therefore, ubiquitous in terrestrial and marine settings (e.g. Zelles 1999; Zhang et al. 2005; Bouloubassi et al. 2006). Fatty acids have previously been identified in geothermal environments, including silica deposits and microbial mats (Shiea et al. 1991; Jahnke et al. 2001; Pancost et al. 2005, 2006; Zhang et al. 2007).

The effect of temperature on the structures and distributions of fatty acids has been well documented. Studies reveal that thermophilic microorganisms tend to increase their average fatty acid chain length (Weerkamp and Heinen 1972; Oshima and Miyagawa 1974; Russell 1984), decrease the degree of unsaturation and degree of branching (Daron 1970; Ray et al. 1971; McElhaney and Souza 1976), and increase the ratio of iso to anteiso branched fatty acids (Shen et al. 1970; Oshima and Miyagawa 1974), in response to elevated temperatures.

**Fig. 6:**
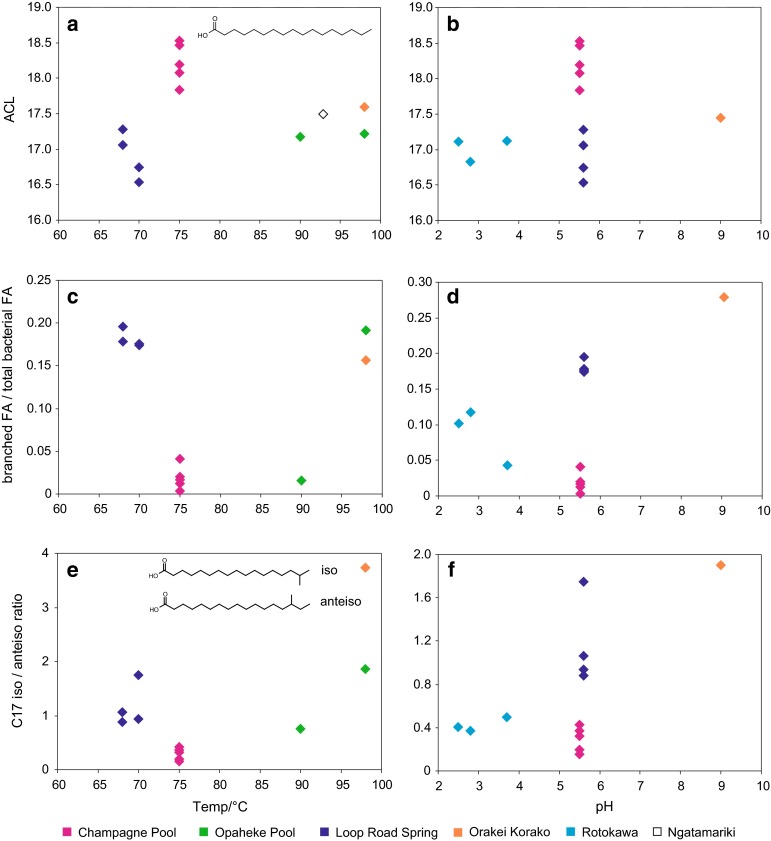
Fatty acid ACL versus **a** temperature and **b** pH; proportion of branched components versus **c** temperature and **d** pH; C_17_ iso to anteiso fatty acid ratio versus **e** temperature and **f** pH. For variations with temperature, samples have been limited to those with pH in the range 5.5–7.2; for variations with pH, samples have been limited to those within the temperature range 68–82 °C

The concentrations and distribution of the fatty acids vary considerably in the sinters analysed. Total concentrations of the bacterial fatty acids (C_14_–C_20_) range from 0.44 in LRa3 to 16.8 mg g^−1^ TOC in OK1 (Table 4). Total fatty acid concentrations are highly variable and show no relationship with temperature or pH (Table 4). Furthermore, no trends in the fatty acid ACL and the proportions of branched and unsaturated fatty acids with temperature and pH are evident (Table 4; Fig. 6a–d). There is a low positive correlation between the C_17_ iso to anteiso ratio and temperature and pH, albeit with low *R* ^2^ values (0.37 and 0.36, respectively) (Fig. 6e, f). Nonetheless, clustering of samples within a particular site is evident (Fig. 6a–f). For example, all Champagne Pool samples exhibit high fatty acid ACLs, whereas sinters from Loop Road springs show significantly lower fatty acid ACLs (Fig. 6a, b), despite the similar temperature and pH conditions of these pools. Furthermore, Loop Road samples have significantly higher proportions of branched fatty acids than those from Champagne Pool (Fig. 6c, d).

**Table 4:** Abundance (mg g^−1^ TOC) and distributions of the fatty acids at each geothermal site

	Temp/°C	pH	Total bacterial FA	ACL	branched/total	unsaturates/total	C15 iso/anteiso	C17 iso/anteiso
CPa1	75	5.5	1.5	18.2	0.020	0.042	0.70	0.20
CPa2	75	5.5	2.2	18.5	0.017	0.030	0.78	0.32
CPa3	75	5.5	2.3	17.8	0.012	0.068	1.4	0.42
CPa4	75	5.5	10.9	18.5	0.003	0.009	1.3	0.15
WT1	75	5.5	–	18.1	0.041	0.040	1.3	0.37
OP2	90	7.2	2.8	17.2	0.016	0.16	1.0	0.75
OP3	98	7.2	0.86	17.2	0.19	0.029	2.0	1.9
LRa1	70	5.6	1.3	16.7	0.18	0.004	5.0	1.8
LRa2	70	5.6	1.3	16.5	0.17	–	2.7	0.93
LRa3	68	5.6	0.44	17.0	0.17	0.11	1.9	1.1
LRa4	68	5.6	0.68	17.3	0.20	0.026	2.3	0.88
OK1	98	7.0	16.8	17.6	0.16	0.060	3.0	3.7
OK1D	78	9.0	–	17.4	0.28	0.006	2.4	1.9
RK1	75	2.8	–	16.8	0.12	0.034	1.2	0.37
RK1F	80	2.5	–	17.1	0.10	0.057	1.4	0.41
RK6A	82	3.7	–	17.1	0.043	0.051	1.6	0.50
NGM-49	93	6.7	–	17.5	–	0.132	–	–

This suggests that the setting and thus the microbial assemblage are the major controls on fatty acid distributions. We argued above that the similarity of biomarkers’ assemblages was evidence for similar microbiological communities at particular hot springs. However, fatty acids are ubiquitous in bacteria and have low biological specificity; thus, the observed distributions likely record the mixing of a much more complex range of bacterial inputs than that recorded by the more taxonomically restricted ether lipids. In this case, even subtle variations in microbial community structure could obscure any effect of temperature and pH on fatty acid composition. Although controls on fatty acid distributions in the geothermal sinters are clearly complex, it is critical to recognise that fatty acid ACLs > 18 are uncommon in mesophilic environments and even geothermal environments characterised by temperatures lower than 50 °C (Shen et al. 1970). Thus, high ACLs, such as those observed at Champagne Pool, are likely due to high temperatures. However, more subtle variations will be difficult to detect and the Loop Road, Rotokawa and Opaheke data clearly indicate that low ACLs are not necessarily indicative of low temperature environments.

#### Hopanoic acids

Hopanoids are pentacyclic triterpenoids and membrane components of a variety of bacteria, including cyanobacteria, methanotrophs and aerobic heterotrophic bacteria (Rohmer et al. 1984; Farrimond et al. 2000), as well as some anaerobic bacteria (Sinninghe Damsté et al. 2004; Fischer et al. 2005). They occur in a range of settings, including geothermal environments, and have been identified in silica sinters and microbial mats (Talbot et al. 2005; Pancost et al. 2005, 2006; Zhang et al. 2007; Gibson et al. 2008, 2014). Previous studies reveal that total hopanoid abundance increases with growth temperature in the thermoacidophilic bacterium *Alicyclobacillus acidocaldarius* (Poralla et al. 1984), the ethanologenic *Zymomonas mobilis* (Schmidt et al. 1986), and an acetic acid bacterium *Frateuria aurantia* (Joyeux et al. 2004). Hopanoids regulate membrane fluidity and have been argued to induce order in the phospholipid membrane (Kannenberg and Poralla 1999), such that higher hopanoid abundances would act to stabilise the membrane at higher temperatures. Joyeux et al. (2004) also reported the biosynthesis of penta-functionalised hopanoids in response to thermal stress in *Frateuria aurantia*, which produced higher amounts of C_31_ hydroxylated hopanoids (derived from pentafunctionalised bacteriohopanoids) at higher growth temperatures. More recently, Welander et al. (2009) demonstrated that hopanoids play a role in maintaining membrane integrity and pH homeostasis in a study of a hopanoid deletion mutant in *Rhodopseudomonas palustris* TIE-1. In this study, the mutant strain, no longer able to produce hopanoids, displayed increased membrane permeability and sensitivity to acidic and alkaline conditions relative to the wild-type strain.

**Fig. 7:**
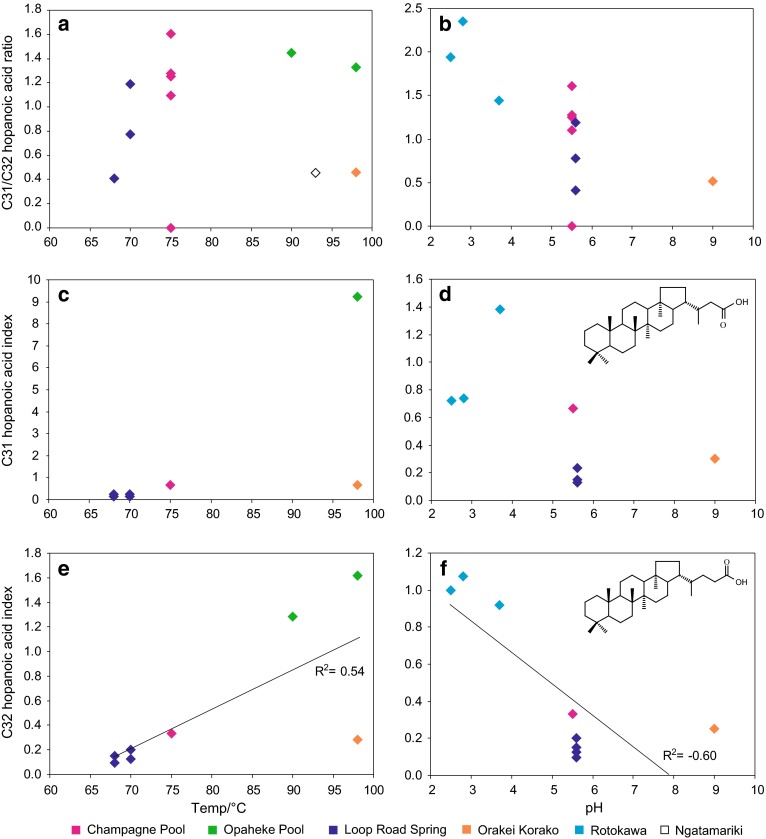
**a** C_31_–C_32_ hopanoic acid ratio versus **a** temperature and **b** pH; C_31_ hopanoic acid index, given by (αβ + βα)/ββ, versus **c** temperature and **d** pH; C_32_ hopanoic acid index versus **e** temperature and **f** pH. For variations with temperature, samples have been limited to those with pH in the range 5.5–7.2; for variations with pH, samples have been limited to those within the temperature range 68–82 °C

A range of hopanoic acids (C_30_–C_34_) was identified in the geothermal silica sinters. These compounds typically derive from the oxidative cleavage of vicinal diols in bacteriohopanoids (Rohmer et al. 1984; Farrimond et al. 2000). Total hopanoic acid abundances are highly variable in the geothermal sinters, ranging from 0.0043 in CPa3 to 1.2 mg g^−1^ (TOC) in OK1 and LRa1 (Table 5). As with the other biomarker classes, concentrations exhibit no relationship with temperature or pH (Table 5). In contrast, their distributions vary significantly between the sinters and in some instances appear to be related to environmental conditions. For example, even though the C_31_/C_32_ hopanoid ratio shows no correlation with temperature (Fig. 7a), at lower pH conditions it is typically higher (Fig. 7b). Given the very different inferred microbial assemblages at Rotokawa, it is difficult to assess whether the higher ratios at lower pH are a result of adaptive responses or a change in community composition.

**Table 5:** Abundance (mg g^−1^ TOC) and distributions of the hopanoic acids at each geothermal site. C_31_ and C_32_ index is given by the (αβ + βα)/ββ ratio

	Temp/°C	pH	Total	C31/C32 ratio	C31 index	C32 index
CPa1	75	5.5	0.009	1.6	–	–
CPa2	75	5.5	–	–	–	–
CPa3	75	5.5	0.004	1.3	–	–
CPa4	75	5.5	0.010	1.1	–	–
WT1	75	5.5	–	1.3	0.67	0.33
OP2	90	7.2	0.016	1.4	–	1.3
OP3	98	7.2	0.054	1.3	9.2	1.6
LRa1	70	5.6	1.2	1.2	0.15	0.20
LRa2	70	5.6	0.23	0.78	0.23	0.13
LRa3	68	5.6	0.19	0.41	0.12	0.15
LRa4	68	5.6	0.15	0.41	0.24	0.10
OK1	98	7	1.2	0.46	0.65	0.28
OK1D	78	9	–	0.52	0.30	0.25
RK1a	75	2.8	–	2.4	0.74	1.1
RK1F	80	2.5	–	1.9	0.72	1.0
RK6A	82	3.7	–	1.4	1.4	0.92
NGM-49	93	6.7	–	0.45	–	–

The hopanoids are also present as a range of stereoisomers in the silica sinters. The biological 17β,21β(H) isomer is typically dominant, but the more thermally stable 17α,21β(H) and 17β,21α(H) isomers are also present and differences in their distributions are evident. The C_31_ and C_32_ indices, defined as the (αβ + βα)/ββ ratio, are both typically elevated at high temperature and low pH (Fig. 7c–f). Although Rosa-Putra et al. (2001) demonstrated the de novo synthesis of 17α,21β(H) hopanoids in *Frankia* strains; in these settings, the thermally stable isomers are likely alteration products of biological precursors. The stereochemical transformation of the biological 17β,21β(H) configuration to the 17α,21β(H) and 17β,21α(H) configurations is directly related to high temperature and low pH conditions (Peters and Moldowan 1991; Pancost et al. 2003). This is consistent with the generally elevated C_31_ and C_32_ indices at higher temperatures and lower pH, demonstrating its potential use as an environmental indicator. Crucially, these transformations are post-mortem chemical changes and, therefore, unlikely to be affected by differences in microbial community structure.

## Conclusions

The abundances and distributions of lipid biomarkers are highly variable in the geothermal silica sinters from the TVZ and can be used to tentatively define microbial community structure. Biomarker profiles reveal three distinct microbial assemblages corresponding to three diverse geochemical environments: the first in the Champagne Pool and Loop Road sinters, the second in the Orakei Korako, Opaheke and Ngatamariki sinters, and the third in the sinters from Rotokawa. Biomarker concentrations appear to have minimal diagnostic value, and we suggest that this is due to processes unrelated to the microbial community, i.e. rates of sinter formation. However, several aspects of the biomarker distributions appear to reflect homeoviscous adaptations to extremes of pH and temperature, whilst others reflect community composition. Specifically, the archaeol to bacterial diether ratio, bacterial diether ACL, degree of cyclisation in the biphytanyl chains of the GDGTs and the C_31_ and C_32_ hopanoic acid indices typically increase with temperature. Archaea typically dominate at high temperatures (Robertson et al. 2005), and studies reveal an increased proportion of cyclic GDGTs (Gliozzi et al. 1983) and increased amounts of the thermally stable 17α,21β(H) hopanoid stereoisomer at higher growth temperatures, consistent with these findings. The changes in diether ACL are a new observation, but are nonetheless consistent with previous lipid studies. Furthermore, the archaeol to bacterial diether ratio, degree of GDGT cyclisation and the C_31_ and C_32_ hopanoic acid indices are typically higher at lower pH. Again, this is consistent with previous findings which report the predominance of archaea (Robertson et al. 2005), and a greater proportion of cyclic GDGTs (Gliozzi et al. 1983) and the 17α,21β(H) hopanoid stereoisomer (Pancost et al. 2003, 2006) at low pH levels. In contrast, bacterial diether ACL generally increases at higher pH, likely reflecting a change in community structure. No trends in fatty acid ACL, and proportions of branched and unsaturated fatty acids with temperature and pH are evident, likely reflecting overprinting due to population influences.

The consistency of many of these results with previous biomarker analyses of pure cultures illustrates that adaptive behaviour in cultured organisms extends to, and can be identified in, complex geothermal communities. Additional proxies based on the post-mortem alteration of hopanoid stereochemistry also appear robust within and between geothermal sites. However, caution is required when interpreting lipid distributions in geothermal environments unless both pH and temperature are constrained. Furthermore, it is clear that the effects of both homeoviscous adaptations and changes in microbial population need to be considered. Nonetheless, this work represents an expanded understanding of the diversity of lipid biomarkers in geothermal environments and reveals the potential use of microbial lipid biomarkers in profiling environmental conditions and microbial community structure.

## References

[CR1] Albers SV, van de Vossenberg J, Driessen AJM, Konings WN (2000). Adaptations of the archaeal cell membrane to heat stress. Front Biosci.

[CR2] Bouloubassi I, Aloisi G, Pancost RD, Hopmans E, Pierre C, Sinninghe Damsté JS (2006). Archaeal and bacterial lipids in authigenic carbonate crusts from eastern Mediterranean mud volcanoes. Org Geochem.

[CR3] Brock TD (1978). thermophilic microorganisms and life at high temperatures.

[CR4] Childs A, Mountain B, O’Toole R, Stott M (2008). Relating microbial community and physicochemical parameters of a hot spring: Champagne Pool, Wai-o-tapu, New Zealand. Geomicrobiol J.

[CR5] Daron HH (1970). Fatty acid composition of lipid extracts of a thermophilic *Bacillus* species. J Bacteriol.

[CR6] DeRosa M, Gambacorta A (1988). The lipids of archaebacteria. Prog Lipid Res.

[CR7] Drici-Cachon Z, Cavin JF, Divies C (1996). Effect of pH and age of culture on cellular fatty acid composition of *Leuconostoc oenos*. Lett Appl Microbiol.

[CR8] Farrimond P, Head IM, Innes HE (2000). Environmental influence on the biohopanoid composition of recent sediments. Geochim Cosmochim Ac.

[CR9] Fischer WW, Summons RE, Pearson A (2005). Targeted genomic detection of biosynthetic pathways: anaerobic production of hopanoid biomarkers by a common sedimentary microbe. Geobiology.

[CR10] Gibson RA, Talbot HM, Kaur G, Pancost RD, Mountain B (2008). Bacteriohopanepolyol signatures of cyanobacterial and methanotrophic bacterial populations recorded in a geothermal vent sinter. Org Geochem.

[CR11] Gibson RA, Sherry A, Kaur G, Pancost RD, Talbot HM (2014). Bacteriohopanepolyols preserved in silica sinters from Champagne Pool (New Zealand) indicating a declining temperature gradient over the lifetime of the vent. Org Geochem.

[CR12] Gliozzi A, Paoli G, De Rosa M, Gambacorta A (1983). Effect of isoprenoid cyclization on the transition temperature of lipids in thermophilic archaebacteria. Biochim Biophys Acta.

[CR13] Guidry SA, Chafetz HS (2003). Siliceous shrubs in hot springs from Yellowstone National Park, Wyoming, USA. Can J Earth Sci.

[CR14] Hazel JR (1995). Thermal adaptations in biological membranes: is homeoviscous adaptation the explanation?. Annu Rev Physiol.

[CR15] Hetzer A, Morgan HW, McDonald IR, Daughney CJ (2007). Microbial life in Champagne Pool, a geothermal spring in Waiotapu, New Zealand. Extremophiles.

[CR16] Hopmans EC, Schouten S, Pancost RD, van der Meer MTJ, Sinninghe Damsté JS (2000). Analysis of intact tetraether lipids in archaeal cell material and sediments by high performance liquid chromatography/atmospheric pressure chemical ionization mass spectrometry. Rapid Commun Mass Spectrom.

[CR17] Huber R, Wilharm T, Huber D, Trincone A, Burggraf S, Konig H, Rachel R, Rockinger I, Fricke H, Stetter KO (1992). *Aquifex pyrophilus* gen. nov. sp. nov. represents a novel group of marine hyperthermophilic hydrogen-oxidizing bacteria. Syst Appl Microbiol.

[CR18] Huber R, Rossnagel P, Woese CR, Rachel R, Langworthy TA, Stetter KO (1996). Formation of ammonium from nitrate during chemolithoautotrophic growth of the extremely thermophilic bacterium *Ammonifex degensii* gen. nov. sp. nov. Syst Appl Microbiol.

[CR19] Jahnke LL, Eder W, Huber R, Hope JM, Hinrichs KU, Hayes JM, Marais DJD, Cady SL, Summons RE (2001). Signature lipids and stable carbon isotope analyses of Octopus spring hyperthermophilic communities compared with those of Aquificales representatives. Appl Environ Microbiol.

[CR20] Jahnke LL, Embaye T, Hope J, Turk KA, Van Zuilen M, Des Marais DJ, Farmer JD, Summons RE (2004). Lipid biomarker and carbon isotopic signatures for stromatolite-forming, microbial mat communities and Phormidium cultures from Yellowstone National Park. Geobiology.

[CR21] Jones B, Renaut RW, Rosen MR (2001). Biogenicity of gold- and silver-bearing siliceous sinters forming in hot (75 °C) anaerobic spring-waters of Champagne Pool, Waiotapu, North Island, New Zealand. J Geol Soc.

[CR22] Joyeux C, Fouchard S, Llopiz P, Neunlist S (2004). Influence of the temperature and the growth phase on the hopanoids and fatty acids content of Frateuria aurantia (DSMZ 6220). FEMS Microbiol Ecol.

[CR23] Kannenberg EL, Poralla K (1999). Hopanoid biosynthesis and function in bacteria. Naturwissenschaften.

[CR24] Kaur G, Mountain BW, Pancost RD (2008). Microbial membrane lipids in active and inactive sinters from Champagne Pool, New Zealand: elucidating past geothermal chemistry and microbiology. Org Geochem.

[CR25] Kaur G, Mountain BW, Hopmans EC, Pancost RD (2011). Relationship between lipid distribution and geochemical environment within Champagne Pool, Waiotapu, New Zealand. Org Geochem.

[CR26] Kaur G, Mountain BW, Hopmans EC, Pancost RD (2011). Preservation of microbial lipids in geothermal sinters. Astrobiology.

[CR27] Konhauser KO, Phoenix VR, Bottrell SH, Adams DG, Head IM (2001). Microbial–silica interactions in Icelandic hot spring sinter: possible analogues for some Precambrian siliceous stromatolites. Sedimentology.

[CR28] Krupp R, Seward TM (1990). Transport and deposition of metals in the Rotokawa geothermal system, New Zealand. Miner Deposita.

[CR29] Langworthy TA, Holzer G, Zeikus JG, Tornabene TG (1983). Iso-branched and anteiso-branched glycerol diethers of the thermophilic anaerobe *Thermodesulfotobacterium*-*Commune*. Syst Appl Microbiol.

[CR30] Macalady JL, Vestling MM, Baumler D, Boekelheide N, Kaspar CW, Banfield JF (2004). Tetraether-linked membrane monolayers in *Ferroplasma* spp: a key to survival in acid. Extremophiles.

[CR31] McElhaney RN, Souza KA (1976). The relationship between environmental temperature, cell growth and the fluidity and physical state of the membrane lipids in *Bacillus stearothermophilus*. Biochim Biophys Acta.

[CR32] Mountain BW, Benning LG, Boerema JA (2003). Experimental studies on New Zealand hot spring sinters: rates of growth and textural development. Can J Earth Sci.

[CR33] Nairn IA, Wood CP, Bailey RA (1994). The Reporoa Caldera, Taupo Volcanic Zone: source of the Kaingaroa Ignimbrites. B Volcanol.

[CR34] Oshima M, Miyagawa A (1974). Comparative studies on the fatty acid composition of moderately and extremely thermophilic bacteria. Lipids.

[CR35] Pancost RD, Bouloubassi I, Aloisi G, Sinninghe Damsté JS (2001). Three series of non-isoprenoidal dialkyl glycerol diethers in cold-seep carbonate crusts. Org Geochem.

[CR36] Pancost RD, Baas M, van Geel B, Damste JSS (2003). Response of an ombrotrophic bog to a regional climate event revealed by macrofossil, molecular and carbon isotopic data. Holocene.

[CR37] Pancost RD, Pressley S, Coleman JM, Benning LG, Mountain BW (2005). Lipid biomolecules in silica sinters: indicators of microbial biodiversity. Environ Microbiol.

[CR38] Pancost RD, Pressley S, Coleman JM, Talbot HM, Kelly SP, Farrimond P, Schouten S, Benning L, Mountain BW (2006). Composition and implications of diverse lipids in New Zealand Geothermal sinters. Geobiology.

[CR39] Pepe-Ranney C, Berelson WM, Corsetti FA, Treants M, Spear JR (2012). Cyanobacterial construction of hot spring siliceous stromatolites in Yellowstone National Park. Environ Microbiol.

[CR40] Perevalova AA, Bidzhieva SK, Kublanov IV, Hinrichs KU, Liu XL, Mardanov AV, Lebedinsky AV, Bonch-Osmolovskaya EA (2010). *Fervidicoccus fontis* gen. nov., sp. nov., an anaerobic, thermophilic crenarchaeote from terrestrial hot springs, and proposal of *Fervidicoccaceae* fam. nov. and *Fervidicoccales* ord. nov. Int J Syst Evol Microbiol.

[CR41] Peters KE, Moldowan JM (1991). Effects of source, thermal maturity, and biodegradation on the distribution and isomerization of homohopanes in petroleum. Org Geochem.

[CR42] Poralla K, Hartner T, Kannenberg E (1984). Effect of temperature and pH on the hopanoid content of *Bacillus acidocaldarius*. FEMS Microbiol Lett.

[CR43] Ray PH, White DC, Brock TD (1971). Effect of temperature on the fatty acid composition of *Thermus aquaticus*. J Bacteriol.

[CR44] Reysenbach AL, Banta A, Civello S, Daly J, Mitchel K, Lalonde S, Konhauser K, Rodman A, Rusterholtz K, Takacs-Vesbach C, Inskeep WP, McDermott TR (2005). Aquificales in Yellowstone National Park. Geothermal biology and geochemistry in Yellowstone National Park: workshop proceedings from the Thermal Biology Institute’s Yellowstone National Park Conference, Oct 2003.

[CR45] Robertson CE, Harris JK, Spear JR, Pace NR (2005). Phylogenetic diversity and ecology of environmental Archaea. Curr Opin Microbiol.

[CR46] Rohmer M, Bouviernave P, Ourisson G (1984). Distribution of hopanoid triterpenes in prokaryotes. J Gen Microbiol.

[CR47] Rosa-Putra S, Nalin R, Domenach AM, Rohmer M (2001). Novel hopanoids from *Frankia* spp. and related soil bacteria. Squalene cyclization and significance of geological biomarkers revisited. Eur J Biochem.

[CR48] Rothschild LJ, Mancinelli RL (2001). Life in extreme environments. Nature.

[CR49] Russell NJ (1984). Mechanisms of thermal adaptation in bacteria—blueprints for survival. Trends Biochem Sci.

[CR50] Schmidt A, Bringer-Meyer B, Poralla K, Sahm H (1986). Effect of alcohols and temperature on the hopanoid content of *Zymomonas mobilis*. Appl Microbiol Biotechnol.

[CR51] Schouten S, Hopmans EC, Pancost RD, Sinninghe Damsté JS (2000). Widespread occurrence of structurally diverse tetraether membrane lipids: evidence for the ubiquitous presence of low-temperature relatives of hyperthermophiles. Proc Natl Acad Sci USA.

[CR52] Schouten S, van der Meer MTJ, Hopmans EC, Rijpstra WIC, Reysenbach AL, Ward DM, Sinninghe Damsté JS (2007). Archaeal and bacterial glycerol dialkyl glycerol tetraether lipids in hot springs of Yellowstone National Park. Appl Environ Microbiol.

[CR53] Schultzelam S, Ferris FG, Konhauser KO, Wiese RG (1995). In situ silicification of an Icelandic hot spring microbial mat: implications for microfossil formation. Can J Earth Sci.

[CR54] Shen PY, Coles E, Foote JL, Stenesh J (1970). Fatty acid distribution in mesophilic and thermophilic strains of the genus *Bacillus*. J Bacteriol.

[CR55] Shiea J, Brassell SC, Ward DM (1991). Comparative analysis of extractable lipids in hot spring microbial mats and their component photosynthetic bacteria. Org Geochem.

[CR56] Simoneit BRT (2002). Molecular indicators (biomarkers) of past life. Anat Rec.

[CR57] Sinensky M (1974). Homeoviscous adaptation—a homeostatic process that regulates the viscosity of membrane lipids in *Escherichia coli*. Proc Natl Acad Sci USA.

[CR58] Sinninghe Damsté JS, Rijpstra WI, Schouten S, Fuerst JA, Jetten MS, Strous M (2004). The occurrence of hopanoids in planctomycetes: implications for the sedimentary biomarker record. Org Geochem.

[CR59] Stetter KO (1996) Hyperthermophiles in the history of life. Ciba F Symp 1–18 9243007

[CR60] Talbot HM, Farrimond P, Schaeffer P, Pancost RD (2005). Bacteriohopanepolyols in hydrothermal vent biogenic silicates. Org Geochem.

[CR61] Thiel V, Peckmann J, Richnow HH, Luth U, Reitner J, Michaelis W (2001). Molecular signals for anaerobic methane oxidation in Black Sea seep carbonates and a microbial mat. Mar Chem.

[CR62] Tobler DJ, Benning LG (2011). Bacterial diversity in five Icelandic geothermal waters: temperature and sinter growth rate effects. Extremophiles.

[CR63] van der Meer MTJ, Schouten S, de Leeuw JW, Ward DM (2000). Autotrophy of green non-sulphur bacteria in hot spring microbial mats: biological explanations for isotopically heavy organic carbon in the geological record. Environ Microbiol.

[CR64] Wagner ID, Wiegel J (2008). Diversity of thermophilic anaerobes. Ann NY Acad Sci.

[CR65] Ward DM, Brassell SC, Eglinton G (1985). Archaebacterial lipids in hot spring microbial mats. Nature.

[CR66] Weerkamp A, Heinen W (1972). Effect of temperature on the fatty acid composition of the extreme thermophiles, *Bacillus caldolyticus* and *Bacillus caldotenax*. J Bacteriol.

[CR67] Weijers JW, Schouten S, Hopmans EC, Geenevasen JA, David OR, Coleman JM, Pancost RD, Sinninghe Damsté JS (2006). Membrane lipids of mesophilic anaerobic bacteria thriving in peats have typical archaeal traits. Environ Microbiol.

[CR68] Welander PV, Hunter RC, Zhang LC, Sessions AL, Summons RE, Newman DK (2009). Hopanoids play a role in membrane integrity and pH homeostasis in *Rhodopseudomonas palustris* TIE-1. J Bacteriol.

[CR69] Zelles L (1999). Fatty acid patterns of phospholipids and lipopolysaccharides in the characterisation of microbial communities in soil: a review. Biol Fert Soils.

[CR70] Zeng YB, Ward DM, Brassell SC, Eglinton G (1992). Biogeochemistry of hot-spring environments. 2. Lipid compositions of Yellowstone (Wyoming, USA) cyanobacterial and Chloroflexus mats. Chem Geol.

[CR71] Zeng YB, Ward DM, Brassell SC, Eglinton G (1992). Biogeochemistry of hot-spring environments. 3. Apolar and polar lipids in the biologically-active layers of a cyanobacterial mat. Chem Geol.

[CR72] Zhang CL, Huang ZY, Cantu J, Pancost RD, Brigmon RL, Lyons TW, Sassen R (2005). Lipid biomarkers and carbon isotope signatures of a microbial (Beggiatoa) mat associated with gas hydrates in the Gulf of Mexico. Appl Environ Microbiol.

[CR73] Zhang CL, Pearson A, Li YL, Mills G, Wiegel J (2006). Thermophilic temperature optimum for crenarchaeol synthesis and its implication for archaeal evolution. Appl Environ Microbiol.

[CR74] Zhang C, Huang Z, Li YL, Romanek C, Mills G, Gibson RA, Talbot HM, Wiegel J, Noakes J, Culp R (2007). Lipid biomarkers, carbon isotopes, and phylogenetic characterization of bacteria in California and Nevada hot springs. Geomicrobiol J.

